# Prognostic factors, patterns of recurrence and toxicity for patients with esophageal cancer undergoing definitive radiotherapy or chemo-radiotherapy

**DOI:** 10.1093/jrr/rrv022

**Published:** 2015-04-23

**Authors:** Matthias F. Haefner, Kristin Lang, David Krug, Stefan A. Koerber, Lorenz Uhlmann, Meinhard Kieser, Juergen Debus, Florian Sterzing

**Affiliations:** 1 Department of Radiation Oncology, Heidelberg University Hospital, Im Neuenheimer Feld 400, Heidelberg 69120, Germany; 2 Heidelberg Institute of Radiation Oncology (HIRO), Im Neuenheimer Feld 400, Heidelberg 69120, Germany; 3 Department of Medical Biometry and Informatics, University of Heidelberg, Im Neuenheimer Feld 305, Heidelberg 69120, Germany; 4 German Cancer Research Center (DKFZ), Clinical Cooperation Unit Radiation Oncology, Im Neuenheimer Feld 280, Heidelberg 69120, Germany

**Keywords:** esophageal cancer, radiotherapy, definitive chemo-radiation, prognostic factors, toxicity

## Abstract

The aim of this study was to evaluate the effectiveness and tolerability of definitive chemo-radiation or radiotherapy alone in patients with esophageal cancer. We retrospectively analyzed the medical records of *n* = 238 patients with squamous cell carcinoma or adenocarcinoma of the esophagus treated with definitive radiotherapy with or without concomitant chemotherapy at our institution between 2000 and 2012. Patients of all stages were included to represent actual clinical routine. We performed univariate and multivariate analysis to identify prognostic factors for overall survival (OS) and progression-free survival (PFS). Moreover, treatment-related toxicity and patterns of recurrence were assessed. Patients recieved either chemo-radiation (64%), radiotherapy plus cetuximab (10%) or radiotherapy alone (26%). In 69%, a boost was applied, resulting in a median cumulative dose of 55.8 Gy; the remaining 31% received a median total dose of 50 Gy. For the entire cohort, the median OS and PFS were 15.0 and 11.0 months, respectively. In multivariate analysis, important prognostic factors for OS and PFS were T stage (OS: *P* = 0.005; PFS: *P* = 0.006), M stage (OS: *P* = 0.015; PFS: *P* = 0.003), concomitant chemotherapy (*P* < 0.001) and radiation doses of >55 Gy (OS: *P* = 0.019; PFS: *P* = 0.022). Recurrences occurred predominantly as local in-field relapse or distant metastases. Toxicity was dominated by nutritional impairment (12.6% with G3/4 dysphagia) and chemo-associated side effects. Definitive chemo-radiation in patients with esophageal cancer results in survival rates comparable with surgical treatment approaches. However, local and distant recurrence considerably restrict prognosis. Further advances in radio-oncological treatment strategies are necessary for improving outcome.

## INTRODUCTION

Esophageal cancer is ranked among the ten most common malignant diseases worldwide. In 2008, there were 482 300 new cases, and 406 800 patients succumbed to their disease [1]. Squamous cell carcinoma (SCC) has been the predominating histology in the past century, but the incidence of adenocarcinoma (AC) of the esophagus and the gastro-esophageal junction is rising in developed countries [2], most likely due to a shift in risk factors [3].

The treatment of esophageal cancer is an excellent example of the introduction of interdisciplinary management approaches in oncology. For patients with locally advanced disease, the addition of neoadjuvant chemo-radiotherapy or peri-operative chemotherapy to surgery can improve locoregional control as well as overall and progression-free survival [4]. There have been four prospective trials [5–8] and a meta-analysis [9] suggesting that results achieved by chemo-radiotherapy alone compared with surgery with or without neoadjuvant therapy seem to be at least equivalent in terms of overall survival (OS), although there was an increased risk of locoregional failure in patients receiving chemo-radiotherapy alone. Only one of the mentioned trials included at least some patients with AC [6], but another randomized trial recruiting patients with AC exclusively found a trend towards improved OS with chemo-radiotherapy compared with induction chemotherapy followed by surgery [10].

Although the introduction of multidisciplinary approaches has improved the outcome of patients with esophageal cancer, comorbidities or frailty preclude the use of combined approaches, especially those involving surgery, in many patients [11, 12]. Furthermore, it has previously been shown that patients with advanced age and comorbidities are underrepresented in clinical trials [13], that these factors are relevant to the clinical outcome [11, 14], and that trial data thus not necessarily represent daily clinical routine [12].

In this article, we present the clinical results of radiotherapy for esophageal cancer and prognostic factors in a large retrospective cohort at a tertiary academic center.

## MATERIALS AND METHODS

Preceding data collection, the study was approved by the institutional ethical review committee.

### Patient population

Patients treated with radiotherapy for esophageal cancer at the Department of Radiation Oncology at the University Hospital Heidelberg and the German Cancer Research Center from 2000 to 2012 were identified from a retrospective database at the National Center for Tumor Diseases (NCT), Heidelberg. Information was gathered on 387 patients. Inclusion criteria for our analysis were met for all patients treated with definitive local radiotherapeutic concepts (curative or palliative) for esophageal cancer (AC or SCC) of any T, N or M stage and any age as sole treatment or in combination with chemotherapy or immunotherapy. Patients with initially neoadjuvant concepts not receiving subsequent surgery due to progression or other reasons were also eligible. Exclusion criteria were neoadjuvant treatment plans with radiotherapy or chemo-radiation followed by surgery, chemotherapy or immunotherapy without irradiation, radiotherapy of metastases, previous or simultaneous malignancies, death before start of planned radiotherapy, or incomplete data. Altogether, 149 patients were excluded. The patient cohort for final analysis encompassed 238 patients.

Data on treatment and toxicity were collected retrospectively from paper and electronic archives at the University Hospital Heidelberg. Toxicity was graded according to the Common Toxicity Criteria for Adverse Events (CTCAE) version 4.

### Treatment

All patients were treated with CT-planned 3D-conformal radiotherapy at the University Hospital Heidelberg or the German Cancer Research Center, Heidelberg. In a minority of patients, intensity-modulated radiotherapy (IMRT), either as step-and-shoot or helical IMRT, was applied. While 74 patients received a total dose of median 50 Gy, 164 patients were treated with a sequential or simultaneous integrated boost up to a median total dose of 55.8 Gy. The median total dose for all patients was 54 Gy, and the median single dose was 1.8 Gy. The radiation field design included the primary tumor site and mediastinal lymphatic drainage 5 cm cranial of the upper and caudal of the lower tumor borders, respectively. Coeliac lymph nodes were included for distantly located tumors, and caudal cervical lymph nodes were included for tumors of the cervical or upper thoracic esophagus. If a boost was indicated, boost volume was defined with margins of 2 cm above and below tumor borders.

Of the 238 patients, 64% received chemotherapy; in over 90% of these, this consisted of combined chemo-radiation, with two cycles of cisplatin (20 mg/m^2^ body surface area (BSA); Days 1–5 and Days 29–33) and 5-FU (1000 mg/m^2^ BSA; Days 1–5 and Days 29–33) followed by another two cycles of cisplatin and 5-FU four and eight weeks after completion of the combined chemo-radiotherapy. In 10% of the cases, mostly in patients with comorbidities precluding the use of cisplatin but with adequate performance status, a combined radio-immunotherapy with cetuximab was applied, with a loading dose of 400 mg/m^2^ BSA one week before the start of radiotherapy and weekly doses of 250 mg/m^2^ BSA thereafter.

### Follow-up

Patients were routinely examined with CT-scan and endoscopy every 3–6 months for the first two years and every 6–12 months thereafter. The median follow-up from the end of radiotherapy was 11.8 months for the entire cohort and 37.5 months for surviving patients.

### Statistics

All survival times were calculated starting from the date of initial diagnosis. OS was defined as the time to death. Progression-free survival (PFS) was defined as the time to local recurrence or occurrence of metastases, depending on which event occurred first. All patients who did not experience the event of interest were censored at the last follow-up date. In univariate analyses, the Kaplan–Meier method was applied to estimate OS and PFS for various group partitions. In univariate and multivariate analyses, a Cox regression model was applied in which *P*-values were determined by Wald-tests. For multivariate analyses, hazard ratios are provided. For all tests, a *P*-value of <0.05 was considered statistically significant. As this was an exploratory analysis, no adjustments for multiple comparisons were performed. The statistical analysis was performed using R (version 3.0.2, R Development Core Team, 2013, URL: http://www.R-project.org/) in combination with the packages ‘splines’, ‘survival’ (version 2.37–7, Therneau, 2014) and ‘xtable’ (version 1.7–1, Dahl, 2013).

## RESULTS

Patient characteristics are listed in Table 1. The median age was 65 years. Over 80% of the patients were male. Most patients suffered from locally advanced disease at the time of diagnosis. About 20% had distant metastases, which consisted mainly of lymph node metastases in the supraclavicular or coelical compartment for cervical and abdominal location of the primary tumor, respectively. Location of the primary tumor (defined by its proximal edge) was cervical in 9.7%, upper thoracic in 27.3%, middle thoracic in 44.9% and lower thoracic/abdominal in 18.1%. The treatment intention was curative in about three-quarters of the cases. Nutritional support with parenteral nutrition or via percutaneous endoscopic gastrostomy was necessary in 2% and 5% prior to radiotherapy. About half of the patients consumed alcohol on a regular basis and/or were current or former smokers.

**Table 1.** Overview: patient characteristics (*n* = 238)

Characteristic		No. (%)
Patient age at diagnosis (years)	Median	65
	Q1–Q3	58–72
Gender	male	197 (82.8)
	female	41 (17.2)
Karnofsky Index (%)	median	85
	Q1–Q3	80–90
Tumor stage	T1	4 (1.7)
	T2	38 (16.0)
	T3	145 (60.9)
	T4	51 (21.4)
Nodal stage (clinical)	N0	44 (18.5)
	N1	139 (58.4)
	N2	47 (19.7)
	N3	8 (3.4)
Metastases	M0	184 (77.3)
	M1 (lymphatic)	25 (10.5)
	M1 (distant)	29 (12.2)
Grading	G1	6 (2.5)
	G2	95 (39.9)
	G3	135 (56.7)
	G4	2 (0.8)
Histology	SCC	193 (81.1)
	AC	41 (17.2)
	Other	4 (1.7)
Chemotherapy	Yes 143 (93.4% Cis/5-FU)	152 (63.9)
	No	86 (36.1)
Immunotherapy (Cetuximab)	Yes	23 (9.7)
	No	215 (90.3)
Total radiation dose (Gy)	median	54
	Q1–Q3	50.4–57.9
Initial hemoglobin (g/dl)	median	12.7
	Q1–Q3	11.4–14.2
Localization (ab ore, cm)	median	27
	Q1–Q3	22–32

### Survival

**Fig. 1.**
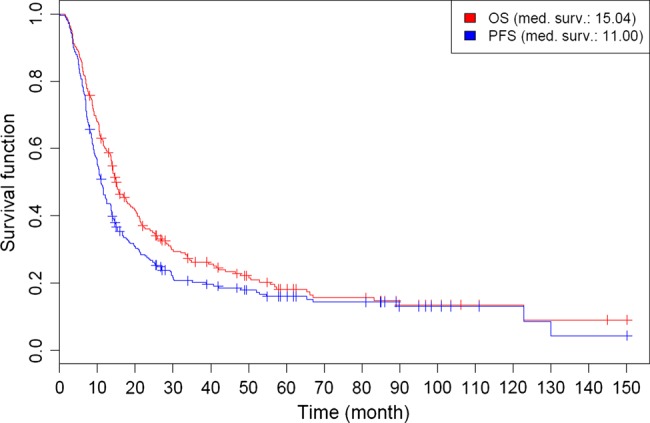
Kaplan–Meier estimates of overall survival (OS) and progression-free survival (PFS) for the entire cohort.

Median OS and PFS for the entire cohort were 15.0 and 11.0 months, respectively (Fig. 1). The estimated 3- and 5-year survival rates were 26.3% and 18.2% for OS and 20.2% and 16.0% for PFS, respectively.

**Fig. 2.**
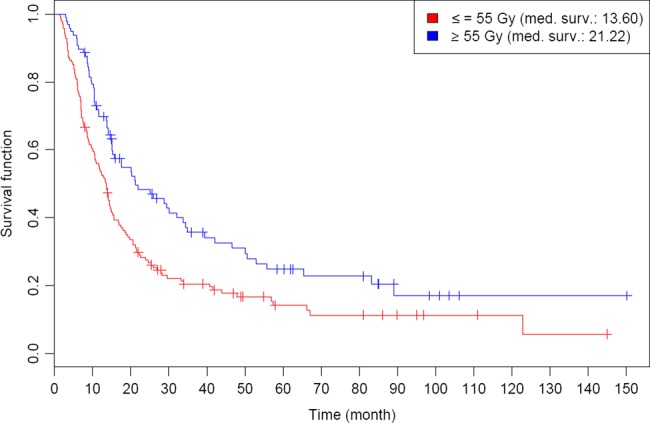
Kaplan–Meier estimates of overall survival (OS) for patients receiving a total dose of ≤55 Gy vs >55 Gy.

Gender and patient age were not associated with OS or PFS. Both tumor and nodal stage had a significant influence on OS, with patients harbouring T1/2 tumors or N0-status achieving a median OS of 25.9 and 29.6 months, respectively. Patients with distant metastases at the initiation of radiotherapy had a dismal prognosis, with a median OS of just 9.8 months. Despite this fact, there were long-term survivors in the M1-subgroup with an estimated 5-year OS of 8.1%. Tumor histology had no significant impact on either OS or PFS, and patients with low tumor grading (G1/2 vs G3/4) had a significantly longer OS (*P* = 0.045). Patients who received a total radiation dose of more than 55 Gy had a median OS of 21.2 months, compared with 13.6 months for patients who received ≤55 Gy (*P* = 0.002), as shown in Fig. 2. However, patients in the lower dose group had a significantly higher prevalence of distant metastases (*P* = 0.034). In the univariate analyses of continuous parameters, we found a significant association of pretherapeutic Karnofsky Index on OS (*P* = 0.02) and PFS (*P* = 0.03) as well as of pretherapeutic hemoglobin on PFS (*P* = 0.009).

**Fig. 3.**
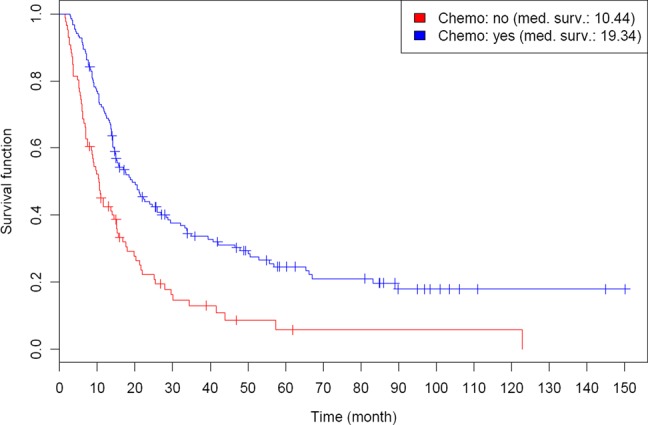
Kaplan–Meier estimates of overall survival (OS) for patients with or without addition of chemotherapy to irradiation.

The univariate analysis of combined treatment revealed that adding cetuximab to sole radiotherapy for patients without chemotherapy added an advantage with respect to OS (with a median survival of 21.6 months vs 8.8 months; *P* = 0.004) and PFS (*P* = 0.03). The benefit for OS was also found in multivariate analysis (MVA), but not for PFS. Furthermore, chemo-radiation was strongly associated with a better OS (*P* < 0.001), with an estimated 5-year OS of 24.6%, compared with 5.8% in patients without chemotherapy (as illustrated in Fig. 3). These results concerning combined treatment as well as the better outcome for higher radiation doses mentioned above were confirmed in MVA. Other prognostic factors with statistical significance in MVA for OS and PFS were T stage and M stage (Table 2).

**Table 2.** Hazard ratios in multivariate analysis (MVA) for (**a**) OS and (**b**) PFS

	a) Hazard ratios for OS		b) Hazard ratios for PFS	
Parameter	Hazard ratio (95% CI)	*P*-value	Hazard ratio (95% CI)	*P*-value
Age	1.00 (0.99, 1.02)	0.701	1.00 (0.98, 1.01)	0.73
Sex (female)	0.77 (0.50, 1.18)	0.228	0.90 (0.60, 1.34)	0.598
Karnofsky Index	0.99 (0.98, 1.01)	0.514	1.00 (0.98, 1.01)	0.575
T3	1.54 (0.95, 2.49)	0.005	1.51 (0.96, 2.36)	0.006
T4	2.43 (1.39, 4.22)		2.32 (1.37, 3.94)	
N1	1.38 (0.89, 2.14)	0.293	1.24 (0.75, 1.87)	0.601
N2/3	1.46 (0.87, 2.46)		1.24 (0.75, 2.03)	
M1	1.56 (1.09, 2.23)	0.015	1.69 (1.19, 2.39)	0.003
G3/4	1.06 (0.78, 1.45)	0.709	1.10 (0.81, 1.49)	0.534
Initial hemoglobin	0.96 (0.89, 1.03)	0.255	0.95 (0.88, 1.02)	0.136
Localization	1.00 (0.98, 1.03)	0.711	1.00 (0.97, 1.02)	0.83
Histologic subtype (AC)	0.86 (0.56, 1.32)	0.499	0.95 (0.62, 1.45)	0.812
Total radiation dose (>55 Gy)	0.68 (0.49, 0.94)	0.019	0.69 (0.51, 0.95)	0.022
Chemotherapy (yes)	0.38 (0.27, 0.54)	<0.001	0.44 (0.31, 0.63)	<0.001
Immunotherapy (Cetuximab) (yes)	0.45 (0.24, 0.83)	0.011	0.61 (0.34, 1.09)	0.097

### Patterns of recurrence

A total of 82 patients (34.4%) experienced locoregional failure. In most of these cases, cancer relapsed locally (84.2%) rather than in regional lymph nodes (15.8%). Further, local failure was mainly observed within the initially irradiated volume (94.2% in-field vs 5.8% out-field). These in-field recurrences were diagnosed after a median period of 12.2 months, and the main part occurred within the first 2 years after first diagnosis (49.2% after 12 months, 81.5% after 24 months). Nearly half of all patients with a locoregional relapse showed distant metastases simultaneously or during further follow-up (48.8%). Almost the same rate of distant failure was observed in the subgroup of local in-field relapses (46.2%).

Salvage treatment strategies mainly included palliative chemotherapy. In the subgroup of locally relapsed tumors (with or without distant spread), the local treatment of choice was palliatively dosed re-irradiation by brachytherapy (16.9%) or small-volume external-beam radiotherapy (7.7%). Extended salvage surgery was only performed in four patients (6.2% and 4.9% in the subgroup of local in-field relapses and all locoregional failures, respectively).

### Toxicity

Data on toxicity during and after radiotherapy are listed in Table 3. Radiotherapy had to be aborted in 8 patients (3.3%) due to toxicity or deterioration of the general condition. A total of 13 patients (5.4%) underwent endoscopic dilation after completion of radiotherapy, in 3 cases due to a tumor recurrence. In 11 patients (4.6%), endoscopic stenting was performed. Thirteen patients (5.4%) underwent endoluminal brachytherapy at the time of local recurrence. During radiotherapy, 6.7% of the patients required parenteral nutrition and 19.2% received nutrition via gastric feeding tube. Six months after completion of radiotherapy, 13.4% of patients still depended on a gastric feeding tube, while 1.3% of patients still received parenteral nutrition.

**Table 3.** Overview: acute, subacute and late side effects with grading

	Acute				Subacute (<6 months)				Chronic (>6 months)			
Grading	G1	G2	G3	G4	G1	G2	G3	G4	G1	G2	G3	G4
**Radiotherapy**	No. (%)	No. (%)	No. (%)	No. (%)	No. (%)	No. (%)	No. (%)	No. (%)	No. (%)	No. (%)	No. (%)	No. (%)
Mucositis	8 (3.4)	12 (5.0)	5 (2.1)	/	1 (0.4)	4 (1.7)	/	/	/	/	/	/
Bleeding	1 (0.4)	/	/	/	1 (0.4)	/	/	/	/	/	1 (0.4)	/
Stricture	/	/	1 (0.4)	/	/	/	10 (4.2)	/	/	/	10 (4.2)	/
Fistula	1 (0.4)	/	/	/	/	2 (0.8)	/	/	/	/	1 (0.4)	/
Xerostomia	4 (1.7)	/	/	/	2 (0.8)	1 (0.4)	/	/	4 (1.7)	1 (0.4)	/	/
Nausea	21 (8.8)	17 (7.1)	7 (2.9)	/	/	/	/	/	/	/	/	/
Dysphagia	34 (14.3)	67 (28.2)	29 (12.2)	1 (0.4)	17 (7.1)	15 (6.3)	5 (2.1)	3 (1.3)	26 (10.9)	23 (9.7)	10 (4.2)	1 (0.4)
Dermatitis	43 (18.1)	17 (7.1)	9 (3.8)	/	6 (2.5)	2 (0.8)	/	/	4 (1.7)	4 (1.7)	/	/
Cardiac toxicity	/	1 (0.4)	/	/	/	/	/	/	/	/	/	/
Pulmonary toxicity	3 (1.3)	/	/	/	1 (0.4)	4 (1.7)	/	/	2 (0.8)	4 (1.7)	1 (0.4)	/
**Chemotherapy**	No. (%)	No. (%)	No. (%)	No. (%)								
Leucopenia	4 (2.6)	15 (9.9)	12 (7.9)	/								
Pancytopenia	1 (0.7)	11 (7.2)	6 (3.9)	/								
Nausea	18 (11.8)	58 (38.2)	23 (15.1)	2 (1.3)								
Emesis	29 (19.1)	31 (20.4)	19 (12.5)	/								
Diarrhea	4 (2.6)	1 (0.7)	2 (1.3)	/								
Edema	2 (1.3)	6 (3.9)	1 (0.7)	/								
Hearing loss	1 (0.7)	2 (1.3)	2 (1.3)	/								

## DISCUSSION

In this retrospective analysis we present a substantial cohort of patients with esophageal cancer from a large oncologic center treated with definitive chemo-radiation or radiotherapy alone. In contrast to most of the pre-existing studies with highly selected patient cohorts, our analysis reflects the actual clinical situation by not excluding T4- or M1-staged patients and thus provides important information about the current status of non-surgical strategies in terms of treatment outcome and tolerability.

In accordance with our results, several other studies have shown that both advanced T stage [5, 8, 15, 16] and irradiation without combined chemotherapy [17–19] are strong indicators for a poor prognosis. This has to be taken into account when evaluating our survival data, as the cohort included >20% of patients with T4 stage or distant metastases, respectively, and approximately one-third received radiotherapy only. With a median OS of 15 months, and 3- and 5-year OS rates of 26.3% and 18.2%, respectively, our results are well in line, even with studies excluding T4- or M1-staged patients [7, 16, 19]. On the other hand, there are other publications reporting slightly better survival rates, probably due to favorable inclusion criteria [5, 8, 20]. Bedenne *et al.* showed a median OS of 19.3% and a 2-year OS of 39.8% for patients treated with chemo-radiation; however, not only T4- or M1-staged patients but also non-responders to the initial treatment phase were excluded [6].

In terms of histopathological parameters, our analysis revealed that high-grade tumors (G3/4) are significantly associated with a worse outcome. Histological subtype did not affect survival, as patients with SCCs showed a median OS of 15.0 months, compared with 15.3 months for those with AC. These findings are in contrast to the assumption that AC is associated with a superior prognosis, based on earlier findings e.g. by Siewert *et al.* [21]. This discrepancy might be caused by a negative selection in our study population, because most of the patients with AC in favorable stages have supposedly been assigned to surgery. Consequently, this instance results in a rather small proportion of ACs in our cohort (17.2%) and a significantly higher incidence of metastatic disease compared with the subgroup of SCCs. However, data showing a better outcome for ACs originate from an era predominantly defined by exclusively surgical approaches. A review of more recent literature taking into account multimodal treatment strategies with perioperative or definitive radio-chemotherapy doesn't show any prognostic difference between AC and SCC [4, 6, 15, 16], confirming the findings of our analysis. The formerly described, prognostic shortcomings of SCCs compared with ACs might have been resolved due to a higher sensitivity to chemo-radiation; for instance, the CROSS trial demonstrated a higher relative benefit of neoadjuvant chemo-radiation for patients with SCC compared with those with AC [22].

Recurring disease frequently limits prognosis and leads to a rapid progression until death. In accordance with other studies [5, 23], treatment failure was mainly due to in-field or distant relapse and rather less often associated to local or regional out-field recurrences. Moreover, in almost half of all in-field recurrences, additional distant metastases were observed simultaneously or during the further course. This fact explains why chemotherapy was the main salvage treatment strategy in our cohort. Extended salvage resection was only performed in a very small subgroup because surgery requires a good general condition, and most patients don't have this at the time of relapse. Hence, less invasive local salvage strategies such as brachytherapy or external beam radiotherapy with small volumes and careful doses were preferred.

To avoid distant failure, many studies have focused on the improvement of systemic treatment options. In accordance with our findings, it has been undoubtedly shown that chemo-radiation is superior to radiotherapy alone [17, 18, 24]. The most commonly used substance scheme is the combination of cisplatin and 5-FU, but other chemotherapeutical regimes in addition to irradiation have shown comparable results, e.g. FOLFOX [25], carboplatin or cisplatin plus paclitaxel [20, 22] or mitomycin c plus 5-FU [19]. However, no groundbreaking improvements have been published over recent years, and further intensification of chemotherapy seems difficult due to toxicity.

It has been shown that esophageal cancer with proof of EGF-R expression (epidermal growth factor receptor) is associated with a worse prognosis [26]. In other entities, EGF-R–positive patients significantly benefit from immunotherapy with e.g. cetuximab [27]. Regrettably, the SCOPE1-trial did not find any prognostic benefit of adding cetuximab to chemo-radiation, but a higher rate of side effects [28]. In contrast, in terms of individual concepts with radiotherapy plus cetuximab monotherapy for patients in rather bad condition or with multiple comorbidities, as an alternative to chemo-radiation we found beneficial effects of immunotherapy on survival, especially OS. However, the rather small number of patients treated with cetuximab alone in our study limits the statistical value of this finding.

Regarding radiation dosage, we were able to show a better survival for patients treated with doses >55 Gy. These findings are well in line with the dose-relationship of other studies [19, 29]. However, there is a bias in favor of the high-dose group, as the subgroup of patients with doses of <55 Gy had a significantly higher incidence of distant metastases, resulting in rather palliative radiation doses to the primary tumor and a worse prognosis in general. A prospective RTOG trial did not find any beneficial effect of dose escalation [30], but there were several protocol violations, and it was stopped unplanned after interim analysis. In summary, there are several indications that there might be a better outcome for higher radiation doses, including our study, but the benefit remains uncertain.

Due to combination with chemotherapy and the inclusion of large mucosal areas, toxicity plays a major role in the evaluation of radiotherapy for esophageal cancer. The most important aspects are mucositis (with individually varying reaction patterns [31]) and consequent malnutrition. Approximately 20–25% of patients treated with chemo-radiation need parenteral nutrition or supportive feeding via gastric tube [5]. Quality of life (QoL) is significantly reduced within the first 6 months after treatment, but returns to pretreatment levels [32]. Further, the decrease is less distinctive compared with surgical approaches [6]. Disregarding dysphagia, high-grade toxicity is mostly assigned to chemotherapy [5, 15, 16]. Special attention has to be given to cervical tumor location, because there is a higher risk of chronic impairment of pharyngeal structures, depending on the applied radiation dose [33, 34].

Cardiac toxicity was very low in our analysis and is very likely underestimated. In large part, this is a methodical limitation of a retrospective analysis, due to the neglect of cardiac events in the follow-up period compared with gastrointestinal or pulmonary issues. Also, because of many early cancer-related deaths, follow-up might be too short for the recording of critical cardiac events and for assessing long-term side effects. In this respect, the prognostic relevance of cardiac toxicity is different compared with cancer patients with a better long-term prognosis, e.g. breast cancer or lymphoma patients. A recent review by Beukema *et al.* showed that cardiac toxicity is a relevant issue in the treatment of esophageal cancer, but also pointed out that current data are insufficient to make prediction models with clinical implications and that there is a need to prospectively assess this problem [35].

Compared with the preceding analysis from our institution [36], with a median OS of 9 months for patients with definitive chemo-radiation, we present a survival improvement, probably because of the introduction of advancing treatment strategies. On the one hand, studies evaluating outcome with reference to treatment year did not find a trend of improving prognosis [16, 37]. On the other hand, a recent meta-analysis has suggested similar outcome for patients with definitive chemo-radiation compared with those with neoadjuvant treatment plus surgery [9]. These results are encouraging and suggest the opportunity for a non-surgical treatment approach as an equivalent, alternative option in general, not only for patients in bad condition or with multiple comorbidities.

In 2014, esophageal cancer is still associated with a poor prognosis, especially in (predominantly prevalent) advanced stages. In the future, further efforts have to be made to improve the prognostic perspective for concerned patients. Advanced diagnostic tools such as PET/CT can help in the acquiring of a more accurate staging [38], give additional information for target volume definition, and may be applied as a prognostic index for treatment response [39]. In addition, therapeutic instruments have to be improved by the adjustment of established techniques [40] or by introducing new modalities, such as particle radiation or new systemic agents. For example, a Phase I/II trial has shown encouraging results for carbon ion radiotherapy [41].

Our study is subject to the well-known limitations of a retrospective analysis. A transfer of our results to the general population should only be made cautiously, taking into account these limitations.

In conclusion, definitive chemo-radiation in patients with esophageal cancer results in admissible survival rates comparable with surgical treatment approaches. Most important prognostic factors are tumor stage, radiation dose and concomitant chemotherapy. However, local and distant recurrence still considerably restrict prognosis. Further advances in treatment have to be provided to improve outcome, and definitive chemo-radiation has to be incorporated into future prospective trials.

## FUNDING

Funding to pay the Open Access publication charges for this article was provided by Deutsche Forschungsgemeinschaft and Ruprecht-Karls-Universität Heidelberg within the funding programme Open Access Publishing.
